# Imaging of Colorectal Liver Metastases: New Developments and Pending Issues

**DOI:** 10.3390/cancers12010151

**Published:** 2020-01-08

**Authors:** Matteo Renzulli, Alfredo Clemente, Anna Maria Ierardi, Irene Pettinari, Francesco Tovoli, Stefano Brocchi, Giuliano Peta, Salvatore Cappabianca, Gianpaolo Carrafiello, Rita Golfieri

**Affiliations:** 1 Radiology Unit, Department of Experimental, Diagnostic and Speciality Medicine, Sant’Orsola Hospital, University of Bologna, 40138 Bologna, Italy; irene.pettinari8725@gmail.com (I.P.); stefano.brocchi85@gmail.com (S.B.); giuliano224@gmail.com (G.P.); rita.golfieri@unibo.it (R.G.); 2 Radiology and Radiotherapy Unit, Department of Precision Medicine, University of Campania “L. Vanvitelli”, 80138 Naples, Italy; alf.clemente@hotmail.it (A.C.); salvatore.cappabianca@unicampania.it (S.C.); 3 Diagnostic and Interventional Radiology, ASST Santi Paolo e Carlo, San Paolo Hospital, 20142 Milan, Italy; amierardi@yahoo.it; 4 Department of Specialised, Experimental and Diagnostic Medicine, Sant’Orsola Hospital, University of Bologna, 40138 Bologna, Italy; francesco.tovoli2@unibo.it; 5 Unit of Radiology, IRCCS Cà Granda, Ospedale Maggiore Policlinico, 20122 Milan, Italy; gcarraf@gmail.com

**Keywords:** metastasis, liver imaging, magnetic resonance imaging, diffusion-weighted imaging, hepatobiliary contrast agent

## Abstract

Computed tomography (CT), magnetic resonance imaging (MRI), and 18-fluorideoxyglucose positron emission tomography (^18^FDG-PET) are historically the most accurate imaging techniques for diagnosing liver metastases. Recently, the combination of diffusion-weighted imaging and hepatospecific contrast media, such as gadoxetic acid in MRI, have been demonstrated to have the highest diagnostic accuracy, sensitivity, and specificity for detecting liver metastases. Various recent meta-analyses have confirmed the diagnostic superiority of this combination (diffusion-weighted imaging and gadoxetic acid-enhanced MRI), especially in terms of per lesion sensitivity, as compared with CT and ^18^FDG-PET, even for smaller lesions (≤1 cm). However, none of the oncological guidelines have suggested the use of MRI as a first-line technique for liver metastasis detection during the staging process of oncological patients. This review analyzes the history of the principal imaging techniques for the diagnosis of liver metastases, in particular of colorectal liver metastases, focusing on the most accurate method (diffusion-weighted imaging combined with gadoxetic acid-enhanced MRI), possible reasons for the lack of its diffusion in the guidelines, and possible future scenarios.

## 1. Introduction

Hepatic metastases represent the most common malignancy of the liver. A focal solid liver lesion is more likely to represent a metastatic tumor than a primary liver malignancy [1]. This explains the great interest of researchers and scientific literature regarding this topic. In fact, in the decade from 2009 to 2018, more than 4700 papers were published on Pubmed using keywords such as “liver metastases AND imaging”.

The vast majority of liver metastases are multiple and, in fact, in only 10% of cases are the liver metastases solitary; moreover, they usually involve various lobes [2]. The liver represents a potential site of metastases from any primary malignant tumor, but the most common metastases involving the hepatic parenchyma come from lung, breast, and gastrointestinal cancers [1,2]. The complexity of both hepatic vascularization and the microenvironment supports the development of cancer cell invasion, permitting increased trapping of tumor cells in the small sinusoids. In particular, the portal vein represents the primary inflow of tumor cell emboli carried in the blood from tumors located in other organs [2,3,4].

The prompt detection of hepatic metastases plays an important clinical role in evaluating the prognosis and in selecting the best treatment strategy. The management of metastatic patients with potentially curative treatments, such as surgical procedures, is based on preoperative imaging. In particular, colorectal liver metastases (CRLM) are very unique in comparison to metastases from other malignancies, because curative resection improves patient survival. Therefore, the precise evaluation before curative treatment is very meaningful in CRLM. Although the final evaluation of hepatic metastatic disease is possible during surgical exploration of the liver, any deviation from the preoperative imaging assessment due to additional intraoperative findings is undesirable.

Computed tomography (CT), magnetic resonance imaging (MRI) and 18-fluorideoxyglucose positron emission tomography (^18^FDG-PET) are historically the most accurate imaging techniques for the diagnosis of liver metastases. However, the preoperative non-invasive assessment of liver metastases has undergone significant changes over the past few decades, opening new scenarios in both diagnostic and surgical approaches. This review analyzes the history of the principal imaging techniques for the diagnosis of liver metastases, in particular of CRLM, focusing on the most accurate imaging methods, such as diffusion-weighted imaging combined with MRI performed with hepatospecific contrast agents, the possible reasons for the lack of its diffusion in the most used and widespread guidelines, and possible future scenarios.

## 2. The History of the Imaging of Hepatic Metastases

When studying the history of the imaging of hepatic metastases over the past 15 years, it was noted that, in three particular years, namely 2005, 2010, and 2016, very important meta-analyses were published [5,6,7,8,9,10,11] regarding the advances made by imaging for the non-invasive diagnosis of liver metastases.

### 2.1. 2005

The first important meta-analysis which investigated the sensitivity of the principal imaging modalities available at that time, such as CT, MRI, and FDG-PET, for the detection of CRLM was in 2005 [5]. The authors demonstrated the significantly higher sensitivity of FDG-PET (94.6%) on a per-patient basis as compared with that of the other imaging modalities, such as non-helical CT (60.2%), helical CT (64.7%) or MRI at 1.5 T (75.8%). However, even then, gadolinium-enhanced MRI revealed the highest sensitivity on a per-lesion basis (78.2%) as compared to CT (71.4%) and FDG-PET (75.9%). Despite these results, FDG-PET became the modality of choice in evaluating hepatic metastases and was considered as part of the preoperative evaluation of resectability in oncological patients [5]. At the same time, according to the majority of international guidelines, CT or MRI could alternatively be chosen to study liver metastases, even though the same guidelines did not report any differences in terms of diagnostic accuracy between CT and MRI [6].

### 2.2. 2010

The year 2010 represented a turning point regarding the issue of the non-invasive diagnosing of hepatic metastases. In fact, in their large meta-analysis, Niekel et al. confirmed the highest sensitivity on a per-patient basis of FDG-PET vs. MRI and CT (94.1%, 88.2%, and 83.6%, respectively) [7]. However, MRI reached the best sensitivity on a per-lesion basis, especially for lesions smaller than 10 mm (80.1%). For these reasons, the authors concluded that the preferred first-line modality for evaluating CRLM should be represented by MRI, while FDG-PET should be used as a second-line modality. 

In the same year, in accordance with the meta-analysis of Niekel et al. [7], Floriani et al. analyzed the per-lesion sensitivity of all the above-mentioned imaging techniques, such as FDG-PET, MRI, and CT in their meta-analysis [8]. In agreement with Niekel et al., Floriani et al. confirmed the highest sensitivity on a per-patient basis of FDG-PET vs. MRI and CT (93.8%, 81.1%, and 74.8%, respectively) and the longstanding belief that MRI showed the same sensibility as FDG-PET (86.3% and 86%, respectively) and a better sensitivity than CT (82.6%; *p* < 0.0001) in per-lesion analysis [8]. Moreover, MRI sensitivity was significantly different when liver-specific contrast agents were administered (86.3%) and the preferential use of MRI for the detection of liver metastases was confirmed.

However, the two meta-analyses published in 2010 presented some differences. The first was the different time period analyzed by the two groups. Niekel et al. [7] carried out a comprehensive search for articles published from January 1990 to January 2010, a very long period in which many technical developments were introduced, probably causing bias. On the other hand, Floriani et al. [8] carried out a search for articles published from January 2000 to August 2008, a very short period. The second difference was the choice of only evaluating papers which used MRI performed with hepatobiliary contrast agents. In fact, Niekel et al. [7] stated that data regarding liver-specific contrast materials, such as gadobenate dimeglumine (Gd-BOPTA, Multihance; Bracco, Princeton, NJ, USA) or gadoxetic acid (Gd-EOB-DTPA, Primovist; Bayer Schering, Berlin, Germany), were unavailable or limited to other focal liver diseases such as hepatocellular carcinoma. Instead, in their analysis of 25 articles, Floriani et al. [8] included eight articles which utilized liver-specific contrast media, such as mangafodipir trisodium (MnDPDP) or resovist (SPIO). Therefore, in the same year (2010), MRI was considered to be the first-line technique in detecting liver metastases according to two different meta-analyses, and, moreover, MRI with hepatobiliary contrast agents was demonstrated to have the best sensitivity in this field according to one meta-analysis. However, MRI did not appear in the majority of the most widely used international oncological guidelines.

The year 2010 and those immediately thereafter represented decisive moments in liver imaging. In fact, on the one hand, robust data demonstrated that MRI with hepatospecific contrast agents was the most sensitive imaging technique in evaluating liver metastases. On the other hand, some hepatospecific contrast media commercially available at that time, such as MnDPDP or SPIO, have since been withdrawn from the market. Fortunately, in the same years, the “second generation” of hepatobiliary contrast agents was diffusely utilized in current clinical practice, continuously changing MRI evaluation of the liver. These new contrast media were Gd-EOB-DTPA and Gd-BOPTA. In particular, as compared with the previous “old” liver-specific contrast agents (i.e., MnDPDP or SPIO), the new hepatobiliary contrast agents furnished both the dynamic phases (arterial, portal, and delayed phases) and the hepatobiliary phase in a single injection, reducing examination time and complications. In 2010, in one of the first published series concerning Gd-EOB-DTPA MRI, Choi et al. demonstrated that this technique had a sensitivity of 90.3% in detecting liver metastases ≤1 cm [9]. As a consequence, MRI performed with the “second generation” of hepatobiliary contrast agents became the modality of choice in clinical practice for the detection of hepatic metastases, surpassing the other dynamic imaging techniques. In 2014, an important randomized multicenter trial (VALUE) compared the diagnostic performance of three different imaging techniques such as Gd-EOB-DTPA MRI, MRI with extracellular contrast medium, and contrast-enhanced CT in patients with suspected CRLM [12]. The study showed the diagnostic superiority of Gd-EOB-DTPA MRI as compared to the other imaging modalities. In fact, additional imaging was required in 0 of 118 patients initially evaluated with Gd-EOB-DTPA MRI, in 19 (17.0%) of 112 patients initially assessed using MRI with extracellular contrast agent, and in 44 (39.3%) of 112 patients primarily evaluated with contrast-enhanced CT (*p* < 0.001). Moreover, the authors demonstrated that confidence in diagnosis and therapeutic decision were high or very high in 98.3% of patients when the initial imaging modality was Gd-EOB-DTPA MRI, significantly superior (*p* < 0.001) to MRI with extracellular contrast medium (85.7%) and contrast-enhanced CT (65.2%) [12]. Furthermore, the information provided by Gd-EOB-DTPA MRI resulted in a decreased number of patients with intraoperative modifications of the established surgical plan in patients undergoing liver resection [12]. The observation that a higher percentage of patients, in the group where Gd-EOB-DTPA MRI was the initial imaging procedure, reached a higher successful treatment was particularly interesting. Perhaps the higher confidence in the preoperative imaging stimulated more audacious treatment decisions, especially regarding more aggressive surgical approaches.

In the same period in which the vast majority of scientific studies was focused on the use of the new second-generation of hepatospecific contrast media, a new MRI technique was also taking hold, namely diffusion-weighted imaging (DWI). Diffusion-weighted imaging measures the degree of diffusion of water molecules in biological tissues in vivo; diffusion represents the random motion of water molecules, also known as Brownian motion; during its motion, each particle collides with those nearby and moves, following an erratic course [13]. The diffusion of water molecules is a biophysical parameter which correlates with the structural characteristics of tissues under both physiological and pathological conditions [13]. The rapid evolution and therefore diffusion of DWI worldwide has led to an important milestone in abdominal radiology and, in particular, in liver imaging. In fact, many papers have evaluated the value of DWI in the detection and characterization of focal liver lesions. In particular, many authors compared the diagnostic performances of DWI-MRI, Gd-EOB-DTPA MRI and the combination of both methods in evaluating liver metastases [14,15,16,17,18]. In a prospective study in 2015, Kim et al. clearly demonstrated that Gd-EOB-DTPA MRI combined with DWI was much more accurate than contrast-enhanced CT (98% vs. 85%, respectively), having important effects in patients with potentially resectable disease [19]. Subsequently, in a prospective study, Schulz et al. confirmed the significantly highest per-lesion sensitivity of combined Gd-EOB-DTPA MRI with DWI with respect to all the other imaging modalities, such as CT and FDG-PET (90%, 68%, and 61%, respectively), especially for lesions <10 mm (74%, 16%, and 9% respectively) [20]. Therefore, these authors [19,20] concluded that the detection of liver metastases should be based on MRI as a first-line imaging technique in order to avoid underestimation of liver involvement and ensure correct management. It is important to underline that the differential uptake of FDG in liver metastases above the liver background update is mainly due to the upregulation of different glucose transporters: GLUT2 in liver parenchyma and different transporters in metastases depending on primary tumors (i.e., metastases from lung and breast cancers are GLUT5 positive while those from colorectal cancer are GLUT3 positive). This aspect will affect the sensitivity of this technique.

### 2.3. 2016

After all these experiences, the year 2016 represents the third and last fundamental step in the imaging of liver metastases. In fact, in 2016, a systematic review and meta-analysis, involving more than 1200 liver lesions evaluated over a 15-year period, confirmed the higher per-lesion sensitivity of Gd-EOB-DTPA MRI as compared to contrast-enhanced CT (median 94.9% vs. 74.2%, respectively; *p* < 0.001) without statistical differences in terms of specificity (median 86.6% vs. 94.1%; *p* = 0.44) [21]. The superiority of Gd-EOB-DTPA MRI over contrast-enhanced CT was also demonstrated for lesions <1 cm (per-lesion median sensitivity of 85.7% and 50%, respectively; *p* < 0.001).

Moreover, in the same year (2016), Vilgraine et al. published another comprehensive meta-analysis in which they included 1989 patients with 3854 hepatic metastases [10]. The authors, assuming the superiority of MRI over other imaging techniques such as CT, analyzed the sensitivity of DWI alone, Gd-EOB-DTPA MRI alone, and a combination of the above techniques (DWI and Gd-EOB-DTPA MRI) for detecting liver metastases on a per-lesion basis. When considered independently, DWI-MRI was less sensitive than Gd-EOB-DTPA MRI for detecting liver metastases (87.1% vs. 90.6%); however, the combination of both techniques demonstrated the highest value of per-lesion sensitivity (95%; *p* < 0.0001). Moreover, the authors found that similar results were observed in articles which compared the three techniques simultaneously, with only CRLM and liver metastases smaller than 1 cm. The conclusion was that in metastatic patients, the combination of DWI and Gd-EOB-DTPA MRI ensured the highest sensitivity for detecting liver metastases on a per-lesion basis.

The economic implications of the initial imaging with MRI in patients with suspected liver metastases have always been a matter of debate in the scientific community. In the majority of cases, this is an argument in favor of the use of CT instead of MRI as the first-line technique, together with the scarce spread of MRI in the world. However, there were no robust scientific evidences on these issues until 2016.

**Figure 1:**
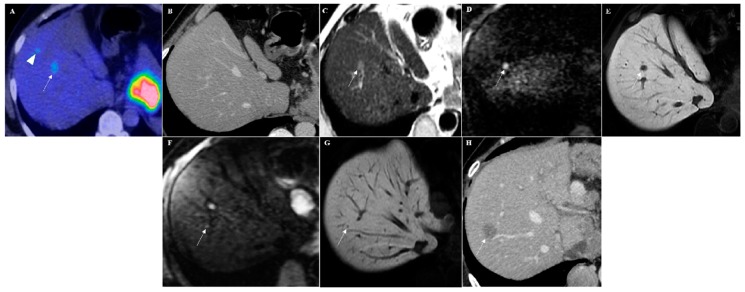
A 61-year-old male with a clinical history of gastric cancer. (**A**) 18-fluorideoxyglucose positron emission tomography (^18^FDG-PET) image shows two focal lesions in the segment VIII of the liver with increased uptake (SUVmax 4.7) (arrowhead and arrow). (**B**) In the axial computed tomography (CT) scan image, no suspicious lesions were visible. Diffusion-weighted imaging (DWI)-Gd-EOB-DTPA magnetic resonance imaging (MRI) examination showed only one slight hyperintense focal lesion on T2-weighted images (arrow in (**C**)) with strong hyperintensity in diffusion-weighted image (arrow in (**D**)) and hypointensity during the hepatobiliary phase (arrow in (**E**)), suspected for a liver metastasis. Moreover, a very small (2 mm) focal lesion with hyperintensity on diffusion-weighted image (arrow in (**F**)) and hypointensity during the hepatobiliary phase (arrow in (**G**)) was also detectable cranially in the same liver segment. An axial contrast-enhanced CT scan performed after six months confirmed this hepatic metastasis (arrow in (**H**)).

In fact, in 2016, Zech et al. assessed the costs of diagnostic workup and surgery for patients with liver metastases from colorectal cancer, comparing three different imaging strategies: Gd-EOB-DTPA MRI, MRI with extracellular contrast media, and contrast-enhanced CT [22]. Surprisingly, the cost of a diagnostic workup in the majority of countries analyzed, including Austria, Germany, Italy, Sweden, Switzerland, and Thailand, was lower when Gd-EOB-DTPA MRI was used as the initial imaging technique as compared with the other strategies. In fact, although Gd-EOB-DTPA MRI was the most expensive imaging modality, no patient in the Gd-EOB-DTPA MRI group required additional imaging examinations in order to reach a decision regarding treatment as compared to 18.1% and 39.7% of the patients in the extracellular contrast media-enhanced MRI and contrast-enhanced CT groups, respectively. Specifically, in the majority of European countries, the diagnostic workup costs were higher due to the need for additional MRI procedures, and the cost of surgery was higher in the Gd-EOB-DTPA MRI group since significantly more patients underwent surgery for a curative approach. Therefore, the superior sensitivity of Gd-EOB-DTPA MRI in detecting liver metastases, the benefits in avoiding additional imaging examinations, and similar diagnostic workup costs suggest that Gd-EOB-DTPA MRI should be the preferred initial imaging modality for evaluating liver resectability in patients with hepatic metastases (Figure 1). In another field of research, such as hepatocellular carcinoma, a study was conducted regarding the economic impact on the management of these patients, using the three different techniques as starting imaging, namely Gd-EOB-DTPA MRI, MRI with extracellular contrast medium, and contrast-enhanced CT [11]. Using a mathematical model, the authors demonstrated that, starting from a similar budget for the three different methods, Gd-EOB-DTPA MRI promoted cost savings, reducing treatments for false positive patients and reducing the requirement for other examinations or biopsies [11]. The main advantage of this approach was that the major part of the money could be used to treat the true positive patients. The authors believe that these results can also be demonstrated in the field of patients with liver metastases in which the use of Gd-EOB-DTPA MRI could reduce unnecessary treatment for false positive patients and could reduce the use of other imaging techniques or biopsies, thus allowing the use of increasingly limited economic resources for the treatment of those patients who could really benefit from treatment, especially for aggressive ones.

### 2.4. From 2016 to Today

What has happened since 2016? Following the improvements in the DWI technique and Gd-EOB-DTPA MRI, additional papers in the last five years have investigated the diagnostic accuracy of this combination in evaluating liver metastases. The results confirmed the diagnostic advantages of this combination in detecting liver metastases, pointing out its superiority as a first-line imaging technique. In particular, Asato et al. confirmed the significantly higher overall sensitivity of combined DWI plus Gd-EOB-DTPA MRI (91.4%) over CT (80.9%), observing a higher sensitivity especially in smaller-sized lesions (73.3% vs. 56.0% for lesions ≤1 cm; 91.9% vs. 80.8% for lesions >1 cm and ≤2 cm; 99.2% vs. 95.7% for lesions >2 cm, respectively) [23].

In 2018, an interesting study investigated the potentiality of Gd-EOB-DTPA MRI in affecting the survival rate in patients with synchronous liver metastases from colon cancer as compared with patients assessed only with CT [24]. The estimated five-year survival rate in the MRI group was significantly superior (70.8%) as compared with that in the CT group (48.1%). Moreover, in the multivariate analysis, the only factors affecting five-year patient mortality were pathologic nodal (pN) staging and evaluation with MRI; no other characteristics of the tumors (i.e., invasion of the resection margin of the primary cancer, median number of metastases, etc.) nor of the patients (i.e., age, sex, previous adjuvant chemotherapy) affected the five-year mortality rate. Therefore, the authors concluded that the additional preoperative evaluation by Gd-EOB-DTPA MRI facilitated more accurate detection of liver metastases and thus a better selection of curative treatment, allowing a better survival rate [24].

Also, in 2018, Choi et al. reported in their meta-analysis a sensitivity for MRI, CT, and PET/CT of 93.1% (95% confidence interval (CI), 88.4–96.0%), 82.1% (95% CI, 74.0–88.1%), and 74.1% (95% CI, 62.1–83.3%), respectively [25]. The majority of the above-mentioned published series were carried out using Gd-EOB-DTPA MRI. Are these results reachable only with the use of this contrast media? Is there the possibility of obtaining the same results with other hepatobiliary contrast agents? In 2019, Zhang et al. evaluated the sensitivity of MRI performed with Gd-BOPTA as compared with MRI performed with Gd-EOB-DTPA for detecting liver metastases [26]. The meta-analysis showed an overall sensitivity of 95.1% and a positive predictive value (PPV) of 90.9% for Gd-BOPTA MRI in detecting metastases, obtaining a comparable diagnostic performance with Gd-EOB-DTPA MRI [26]. These results confirmed that the hepatobiliary phase improved the detection of liver metastases when compared to other imaging techniques regardless of the type of hepatobiliary contrast agent used.

Table 1 summarize the sensitivity values of all the imaging techniques reported in the meta-analysis previously analyzed.

## 3. Impact of the New Imaging Novelties on International Guidelines

**Figure 2:**
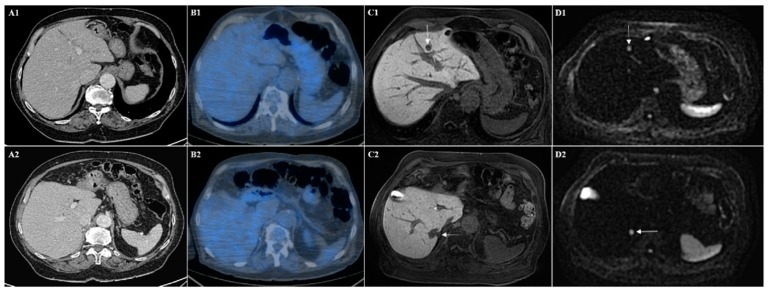
(**A**) CT and (**B**) PET-CT images show the absence of liver metastases in different liver section (1 and 2). (**C**) Hepatobiliary phase of Gd-EOB-DTPA MRI and (**D**) diffusion-weighted imaging show in the same patient the presence of two liver metastases from sarcoma in two different levels, one in the segment III (arrows in (**C1**) and (**D1**)) and one in the segment VII (arrows in (**C2**) and (**D2**)).

The development of MRI with hepatobiliary contrast agents, such as extracellular contrast agents, permits the acquisition of contrast-enhanced liver images during the dynamic phases and in the delayed hepatobiliary phase, after specific uptake of the contrast media by the hepatocytes. As analyzed above, the hepatobiliary phase plus DWI has clearly been demonstrated to improve the detection of liver metastases when compared to all other imaging modalities (both CT and FDG-PET images) (Figure 2) with important implications regarding patient survival and medical costs [10,11,22,23,24,25,26]. What impact has this knowledge had on the most important international oncological guidelines? Unfortunately, they do not seem to have had any relevance regarding the current clinical guidelines, especially regarding the management of gastrointestinal cancer. In fact, in the most recent edition of the European Society for Medical Oncology (ESMO) consensus guidelines for the treatment of patients with metastatic colorectal cancer (revised in 2017), MRI was considered to be a second-line imaging modality for assessing liver metastases following CT examination and was considered to be at the level of liver ultrasound or PET-CT [27]. In fact, in “Recommendation 11: imaging in the identification and management of disease”, the authors state that: “Imaging should first comprise abdominal/pelvic and thoracic CT scans and, in the case of doubt, a second method such as ultrasound (contrast enhanced- ultrasound), MRI or PET/CT scan depending on the location of the metastases. Ultrasound may be helpful to characterize liver metastases, MRI liver, peritoneal or pelvic metastases and PET/CT extrahepatic disease (IV, B)”. Interestingly, there was no mention of hepatospecific contrast media in the entire manuscript.

Furthermore, in the current ESMO guidelines for rectal cancer diagnosis treatment and follow-up, published in 2017, MRI is considered to be the first-line imaging method for loco-regional tumor staging while CT is preferred as the technique of choice for the assessment of distant metastases [28]. Curiously, the level of evidence assigned to their imaging recommendation is quite poor (Level V: studies without a control group, case reports and expert opinions). (No) Interestingly, there was also no mention of hepatospecific contrast media in this entire manuscript.

In the colorectal cancer guidelines of the Association of Coloproctology of Great Britain and Ireland (ACPGBI) published in 2017, CT was considered the modality of choice for cancer staging and MRI assumes a role only in the characterization of equivocal liver lesions [29].

At radiological congresses, it is often said that the guidelines are written by clinicians and, for this reason, the newest findings which imaging offers are not immediately reported in these papers. Therefore, the authors wonder if something would change even if the guidelines were written by radiologists. Unfortunately, there were no differences in the imaging approach for colorectal metastatic disease evaluation in the Appropriateness Criteria—Pretreatment Staging of Colorectal Cancer drafted by the American College of Radiology (ACR) in 2017 [4]. They stated that, despite differences in locoregional staging between colon and rectal cancer, the evaluation of distant metastases has the same approach; “it is difficult to determine the best imaging modality for patients with CRLM” [29]. Moreover, they specified that “the available evidence supports that both MRI and CT detect liver lesions with high accuracy” [4].

The lastest versions of the National Comprehensive Cancer Network (NCCN) guidelines for colon and rectal cancers state that CT remains the first-line imaging technique [30,31]. However, NCCN guideline suggest that if liver-directed therapy or surgery is contemplated, a hepatic MRI with intravenous routine extra-cellular or hepatobiliary contrast agent is preferred over CT (and PET/CT) to assess the exact number and distribution of metastatic foci for local treatment planning.

## 4. Future Scenarios

The introduction of hepatobiliary contrast media into MRI has yielded important information not only in lesion detection and characterization but also concerning the entire hepatocyte uptake. In particular, many additional findings can be obtained during the hepatobiliary phase, especially regarding chemotherapy-induced liver disease. The early detection of chemotherapy-induced sinusoidal obstruction syndrome (SOS) is an interesting topic. Previously known as veno-occlusive disease, SOS is a distinctive and potentially fatal condition of hepatic injury which occurs predominantly after drug or toxin exposure; SOS can be considered to be an adverse side effect in patients with CRLM treated with oxaliplatin [32,33]. Currently, Gd-EOB-DTPA MRI is the best imaging technique for assessing this negative side effect [32]. In fact, the depiction of diffuse reticular hypointensity in the background liver, visible during the hepatobiliary phase, represents the key finding of the disease. It can be explained by the reduction in the organic anion transporting polypeptide 1B3 (OATP1B3) in the centrilobular hepatocyte, with effects resulting from the damage of the centrilobular hepatocytes [32]. This finding has been increasingly observed in chemotherapy-associated liver injuries. Therefore, Gd-EOB-DTPA may be a very useful tool in detecting and depicting SOS at a relatively early stage before the vascular manifestations detected by imaging and, therefore, before its clinical manifestation [32]. This finding has serious clinical implications. The association between the presence of SOS and increased postoperative morbidity has suggested that sinusoidal injury is associated with a significantly higher complication rate and poorer liver functional reserve among patients undergoing a major hepatectomy. This indicates the importance of a careful surgical candidate selection in patients with liver metastases previously exposed to oxaliplatin-based treatment [33]. Moreover, patients with liver metastases showing SOS during the hepatobiliary phase (appearing as reticular hypointensity) demonstrated a higher two-year progression rate (33.2%) as compared to those who did not show reticular hypointensity of the liver parenchyma during the hepatobiliary phase (4.9%) [34]. These results suggest that the evaluation of liver disease requires special care in patients with liver metastases since the possibility of early detection of SOS could change the therapeutic strategy and impact prognosis. In future directions, there are new emerging technologies, such as MR fingerprinting which makes MRI quantitative and there are molecularly and/or cellularly targeted radio-ligands for PET imaging.

Waiting for robust evidences from these new emerging techniques, it is necessary to have some data in order to understand the importance of the correct diagnosis of liver metastases using the standard imaging methods. For example, the most common sites of metastatic involvement in colorectal cancer are the liver and the lungs. Approximately 14.5% of patients present synchronous liver involvement, and the five-year cumulative metachronous liver metastasis rate is 14.5% (3.7% for stage I, 13.3% for stage II, and 30.4% for stage III (*p* < 0.001)). On the one hand, the current paradigm of treatment is to remove all liver metastases if feasible because survival for patients with liver metastases is <1% at five years. Hence, the accurate depiction of the size, distribution, and number of liver metastases is the primary goal of staging [2,3,4]. On the other hand, in a consecutive medicolegal necropsy series, benign hepatic tumors and tumor-like conditions, excluding liver cysts, occurred in 52% of males, having an increasing incidence with age [35,36]. Therefore, when studying oncological patients, it is necessary to utilize an imaging technique which can not only correctly identify all the lesions but also correctly differentiate benign from malignant lesions. The combination of Gd-EOB-DTPA MRI and DWI could become an essential imaging tool in evaluating liver disease as in other fields of imaging, such as that of primary malignancies [37,38,39]. Recent technical developments have certainly contributed to increasing its diagnostic performance in the detection and characterization of liver malignancy, demonstrating a notable impact on patient outcome and medical costs. Additional efforts should be made in clinical research to definitively establish the role of MRI as a first-line imaging technique in order to increase its role in current oncological practice, and additional effort should also be made to introduce these developments into current clinical practice.

## 5. Conclusions

In conclusion, the combination of Gd-EOB-DTPA MRI and DWI has become the most accurate imaging modality in the evaluation of colorectal liver metastases. Additional effort should be made to introduce the developments of imaging of colorectal liver metastases into current clinical practice.

## Figures and Tables

**Table 1:** Per-lesion sensitivity of EOB-MRI, Gd-MRI, CT, and PET/CT for diagnosing colorectal liver metastasis.

Author (Year of Publication)	Number of Included Studies	Gd-EOB-DTPA-MRI Per-Lesion Sensitivity (%)	Gd-MRI Per-Lesion Sensitivity (%)	CT Per-Lesion Sensitivity (%)	PET-CT Per-Lesion Sensitivity (%)
Bipat (2005)	61	-	78.2	71.4	75.9
Floriani (2010)	25	-	86.3	82.6	86
Niekel (2010)	39	-	80.3	74.4	81.4
Vreugdenburg (2016)	11	94.9	-	74.2	-
Vilgrain (2016)	39	95 *	87.1 ^§^	-	-
Choi (2018)	24	93.1	-	82.1	74.1
Zhang (2019)	10	95.1 ^∫^	88.1	-	-

Articles are listed according to the year of publication and in alphabetical order according to the names of the first authors within the same year of publication. MRI: magnetic resonance imaging; Gd-EOB-DTPA-MRI: MRI performed with gadoxetic acid; Gd-MRI: MRI performed with gadolinium; CT: computed tomography; PET: positron emission tomography. * This value refers to the sensitivity of Gd-EOB-DTPA MRI plus diffusion-weighted imaging. ^§^ This value refers to the sensitivity of Gd-MRI plus diffusion-weighted imaging. ^∫^ This value refers to the sensitivity of MRI performed with gadobenate.
